# MRP1-Collateral Sensitizers as a Novel Therapeutic Approach in Resistant Cancer Therapy: An In Vitro and In Vivo Study in Lung Resistant Tumor

**DOI:** 10.3390/ijms21093333

**Published:** 2020-05-08

**Authors:** Chiara Riganti, Roberta Giampietro, Joanna Kopecka, Costanzo Costamagna, Francesca Serena Abatematteo, Marialessandra Contino, Carmen Abate

**Affiliations:** 1 Department of Oncology, University of Turin, Via Santena 5/bis, 10126 Turin, Italy; chiara.riganti@unito.it (C.R.); joanna.kopecka@unito.it (J.K.); costanzo.costamagna@unito.it (C.C.); 2 Department of Pharmacy-Drug Sciences, University of Bari “A. Moro”, Via Orabona 4, 70125 Bari, Italy; giampietroroberta@gmail.com (R.G.); francesca.abatematteo@uniba.it (F.S.A.); carmen.abate@uniba.it (C.A.)

**Keywords:** collateral sensitivity, MDR, MRP1, resistant tumors, lung tumors, ROS

## Abstract

Multidrug resistance (MDR) is the main obstacle to current chemotherapy and it is mainly due to the overexpression of some efflux transporters such as MRP1. One of the most studied strategies to overcome MDR has been the inhibition of MDR pumps through small molecules, but its translation into the clinic unfortunately failed. Recently, a phenomenon called collateral sensitivity (CS) emerged as a new strategy to hamper MDR acting as a synthetic lethality, where the genetic changes developed upon the acquisition of resistance towards a specific agent are followed by the development of hypersensitivity towards a second agent. Among our library of sigma ligands acting as MDR modulators, we identified three compounds, **F397**, **F400**, and **F421,** acting as CS-promoting agents. We deepened their CS mechanisms in the “pure” model of MRP1-expressing cells (MDCK-MRP1) and in MRP1-expressing/drug resistant non-small cell lung cancer cells (A549/DX). The in vitro results demonstrated that (i) the three ligands are highly cytotoxic for MRP1-expressing cells; (ii) their effect is MRP1-mediated; (iii) they increase the cytotoxicity induced by cis-Pt, the therapeutic agent commonly used in the treatment of lung tumors; and (iv) their effect is ROS-mediated. Moreover, a preclinical in vivo study performed in lung tumor xenografts confirms the in vitro findings, making the three CS-promoting agents candidates for a novel therapeutic approach in lung resistant tumors.

## 1. Introduction

Drug resistance, either acquired or intrinsic, is the major hurdle in treating cancer by chemotherapy. The simultaneous resistance to structurally unrelated drugs, also named multidrug resistance (MDR), often results from the overexpression of some drug efflux pumps in the cell membrane that reduce the intracellular drug levels to less than therapeutic concentrations [1,2,3,4,5,6,7,8]. In particular, three proteins belonging to the ATP-binding cassette (ABC) transporter superfamily have been identified as involved in MDR: P-glycoprotein (P-gp/ABCB1), multidrug resistance-associated Protein 1 (MRP1/ABCC1), and breast cancer resistance protein (BCRP/ABCG2).

During recent decades, several strategies to overcome MDR in cancer have been described. One of the most studied strategies was focused on the inhibition of the pumps through small molecules; inhibitors of MDR pumps, devoid of intrinsic toxicity, upon co-administration with the cytotoxic drugs would hamper the drug efflux and reverse resistance [9,10,11]. However, translation into the clinic of this approach has failed so far. One of the lately proposed approaches is related to a phenomenon called collateral sensitivity (CS): some compounds are able to selectively kill MDR cells but not the drug-sensitive parental cells from which they are derived [12,13,14,15,16]. The term CS was firstly described by Szybalski and Bryson in 1952 after the observations that drug-resistant *Escherichia coli* displayed hypersensitivity to unrelated drugs, thus acquiring a potentially exploitable weakness as a result of the drug selection process [13]. In 1985, the same phenomenon was observed for cells overexpressing P-gp. CS is not yet fully understood and four main hypotheses have been proposed as the inducing mechanisms in MDR cells: (1) increase of reactive oxygen species (ROS) through the induction of futile hydrolysis of ATP; (2) increased sensitivity to changes in energy levels; (3) extrusion of endogenous substrates essential for cell survival; and (4) membrane perturbation [14].

CS can be quantitatively assessed in vitro by determining the selectivity ratio (SR), that is, the ratio between the cytotoxicity of a compound against the parental cell line and its cytotoxicity against the MDR-derived line (SR = EC_50_ parental cells/EC_50_ MDR cells) [14]. When the SR >1, the compound displays higher cytotoxicity against the MDR-derived line than its parental cell counterpart, thus eliciting CS. Conversely, a SR < 1 indicates a resistance of the MDR line towards the compound, likely because the compound is an ABC transporters substrate. When the SR >2, the compound is addressed as a CS-promoting agent [12]. The therapeutic approach based on the CS of cancer cells overexpressing the ABC transporters is substantially different from the strategy of transporter inhibition. The co-administration of an ABC transporters inhibitor with a cytotoxic drug would re-sensitize the MDR cells of the drug to the same level as the cells without a transporter expression. By contrast, treatment with a CS agent results in a more potent action in the MDR cells than in the non-MDR counterparts. The non-MDR cells could then be re-sensitized to the “traditional” cytotoxic drug. CS can be considered as a type of synthetic lethality, where the genetic changes developed upon the acquisition of resistance towards a specific agent are followed by the development of hypersensitivity towards a second agent [14].

Therefore, CS-promoting agents may be used as single agents for MDR tumor treatments, as well as agents re-sensitizing MDR tumors to the commonly employed drugs, killing selectively ABC transporters-expressing cells and/or reducing ABC transporters’ expressions/activity in resistant tumors.

While research of CS agents has been mostly directed among P-gp substrates, whose CS may be determined by an ROS increase due to the ATP consumption responsible for the drug efflux, verapamil (L-type calcium channel antagonist, commonly used for the clinical treatment of hypertension) was found to be a CS agent also in MRP1-expressing cells, exploiting the MRP1 ability to efflux glutathione (GSH) [17]. GSH sustains the intracellular redox status, acting as a redox regulator, cofactor, substrate, and antioxidant [14,18,19,20,21,22,23,24]. The increase in GSH efflux leads to a dysregulation of the redox state of cells with a deep impact on cells’ viability.

Therefore, the modulation of intracellular GSH levels through MRP1 can be a powerful approach in cancer therapy mediated by a sensitizer. In fact, verapamil, after binding to MRP1, is not transported by this pump, but stimulates the MRP1-mediated GSH efflux [20]. The fast and huge GSH extrusion triggers a selective apoptosis of cells overexpressing MRP1, as evidenced by treating MRP1-transfected baby hamster kidney 21 (BHK-21) cells and their parental counterparts (BHK-21) in the same conditions, thus confirming the need of MRP1 in this phenomenon [25,26,27].

**Figure 1:**
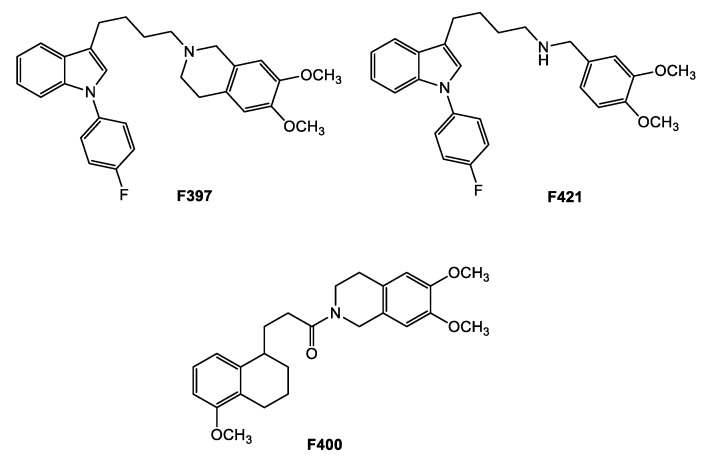
MRP1 modulators from the sigma-2 receptor library.

Nevertheless, very few of the studies focused on CS in MRP1-expressing cells compared with the experimental works focused on CS in P-gp-expressing cells [16,28,29,30]. Due to the impact of MRP1 overexpression in cancer development and drug resistance, and the increasing evidence of its role in CS [19,22,31,32], the aim of the study is the identification of new CS-promoting agents having as the target the MDR protein MRP1. With this in mind, we had to identify proper MRP1 inhibitors, first. Although a number of MRP1 inhibitors have been produced during the past 20 years, they are rarely specific [19,22]. Therefore, we gathered inspiration from our library of sigma-2 receptors’ ligands, that have been recently associated with CS [28,29,33,34]. Sigma-2 receptors, lately identified as TMEM97 proteins, are overexpressed in diverse types of cancers and their modulation leads to cell death upon the activation of a number of still investigated pathways [35,36,37,38,39,40,41,42,43,44,45]. Several sigma-2 receptor high-affinity ligands have been developed during recent decades (and lately reviewed), revealing cytotoxic activity through pathways that appear to be molecule-dependent and cell-type-dependent, with mechanisms that span from caspase-dependent apoptosis, autophagy, increase in ROS, and mithocondrial superoxide production [36,37,40,44]. Some sigma-2 ligands were surprisingly found to be able to kill doxorubicin-resistant cells more than the non-resistant counterparts (breast cancer cells MCF7/DX over MCF7 cells, non-small cell lung cancer A549/DX over A549 cells, colon cancer HT29/DX over HT29 cells) [28,29,33]. The investigation of this property led to find that these compounds were also P-gp substrates and the activation of a futile ATP cycle with an increase in ROS production could be responsible for their CS properties. Herein, we investigated the interaction of these compounds with MRP1 pumps in order to identify novel MRP1 modulators to be exploited as CS agents. Three compounds, derived from three different classes of sigma-2 receptor ligands (Figure 1: **F397**, 6,7-dimethoxy-2-{4-[1-(4-fluorophenyl)-1*H*-indol-3-yl]butyl}-1,2,3,4-tetrahydroisoquinoline [28]; **F421** (*N*-{4-[1-(4-fluorophenyl)-*1H*-indol-3-yl]butyl}-3,4-dimethoxybenzylamine [41]; and **F400** 6,7-dimethoxy-2-[3-(5-methoxy-1,2,3,4-tetrahydronaphthalen-1-yl)propionyl]-1,2,3,4 tetrahydroisoquinoline) [42] emerged as MRP1 modulators in the preliminary screening and for this reason they were further evaluated in the appropriate MDR cell line models and in a preclinical in vivo model. 

## 2. Results and Discussion 

### 2.1. MRP1 Activity and Antiproliferative Activity in MDCK-WT and MDCK-MRP1: Determination of the Selectivity Ratio (SR)

Within the search for novel CS agents acting on MRP1 proteins, the compounds selected among the different series of sigma-2 ligands were investigated for their activity at MRP1, paralleled with their cytotoxicity in the parental Madin–Darby canine kidney (MDCK-wt) cells and in the MDR-derived cells (MDCK-MRP1) for the determination of their SR (EC_50_ parental cells/EC_50_ MDR cells). The activity at the MRP1 pump was determined measuring the transport inhibition of the pro-fluorescent probe Calcein-AM, an MRP1 substrate, in the MRP1-overexpressing cell line (MDCK-MRP1) by the in vitro biological assay usually performed to study an MRP1 interaction. As depicted in Table 1, compounds **F397** and **F421**, bearing the 1-(4-fluorophenyl)indol-3-yl moiety, as in the sigma-2 reference compound siramesine [43,44], together with compound **F400**, belonging to the tetralin series (obtained from SAfiR studies on the sigma-2 reference compound PB28 [42]), emerged as the most promising CS agents among the tested ligands with an SR > 2. In detail, as reported in Table 1, the three compounds **F397**, **F400** and **F421** showed a moderate activity vs. MRP1 (EC_50_ = 16.7, 17.6, and 28 μM, respectively) performed in the MDCK-MRP1 cells but a high collateral sensitivity action displaying an SR = 3.41, 5.91, and 2.47, respectively. The observed CS effect seems due, considering the inhibition of calcein transport activity, to the interaction of the three ligands with MRP1. Moreover, these values are comparable to those observed for the CS reference compound verapamil (SR = 4.7). All the other compounds, belonging to our library of sigma-2 ligands, even if displaying a higher MRP1 activity, did not exert CS-action, as they displayed an SR ≤ 1 (data not shown), similarly to the sigma-2 reference compound siramesine (SR = 0.56). The SR was measured as the EC_50_ of the MDCK cells/EC_50_ of the MDCK-MRP1 cells.

### 2.2. Collatateral Sensitivity Study 

**Figure 2:**
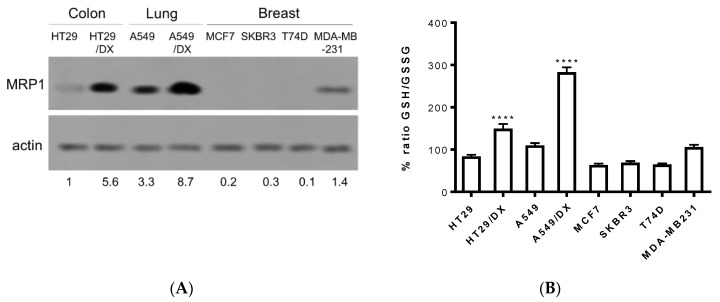
MRP1 expression checked by Western blotting in different cancer cell lines: A549/DX > HT29/DX > A549 > HT29 = MDA-MB-231 > MCF7 = SKBR3 = T74D (**A**): Histogram depicting GSH/GSSG basal levels: A549/DX > HT29/DX > A549 > HT29 = MDA-MB-231 > MCF7 = SKBR3 = T74D (*n* = 3/cell line). Relative quantitations are reported below the immunoblot. (**B**): One-way analysis of variance (ANOVA) analysis: **** *p* < 0.0001 for HT29/DX vs. control HT29, and A549/DX vs. A549.

In order to confirm the entity of the three compounds **F397**, **F421,** and **F400** as sensitizers towards MRP1, further experiments were conducted on cancer cell lines that endogenously express this efflux pump. Thus, observing the results drawn from different studies regarding the MRP1 expression in several solid tumors, we screened the pump’s expression in a variety of cancer cell lines such as the human colon cancer HT29 cell line and the multidrug-resistant counterpart HT29/DX, human non-small cell lung cancer A549 cell line and the multidrug-resistant counterpart A549/DX, and four different types of human breast cancer cell lines: MCF7, SKBR3, T74D, and MDA-MB-231. The immunoblotting study revealed a high amount of MRP1 in both the multidrug-resistant lung and colon cancer cell lines with the highest prevalence in A549/DX, followed by the respective parental cell lines A549 and HT29, and by the MDA-MB-231 cell line (Figure 2A). 

Subsequently, taking into consideration the important role that MRP1 plays in glutathione homeostasis and in the efflux of several chemotherapeutic drugs conjugated with glutathione (PMID:11902585), the basal levels of GSH/GSSG have been evaluated in these cancer cell lines. Hence, as depicted in Figure 2B, all cell lines exhibited similar values of the GSH/GSSG ratio, with A549/DX displaying the highest GSH/GSSG ratio. This result was not surprising. Indeed, A549 cells are known for having high levels of GSH, caused mainly by the high levels of anti-oxidant enzymes [46] that prevent the oxidation of GSH. Moreover, we previously demonstrated that cells with acquired resistance to doxorubicin increase the metabolic flux through the pentose phosphate pathways (PPP) that produce NADPH, a critical metabolite to regenerate the reduced form of glutathione. This phenotype is peculiar for drug-resistant MRP1-expressing cells [47], as A549/DX are. The high levels of GSH may protect cells from the oxidative damage induced by cis-Pt [48] and activate the glutathione S-transferase (GST) enzymes that conjugate GSH to cis-Pt and promote its efflux via MRP1 [49].

Therefore, A549/DX cells were selected as a model to investigate the CS properties of our compounds. Firstly, in order to confirm the data observed on the “pure” model MDCK-MRP1, we tested, in the same experimental conditions, the SR of **F397**, **F400,** and **F421** by measuring their cytotoxicity in the resistant A549/DX cells and their parental counterpart A549, obtaining an SR ≥ 2 for all (SR = 2.5 for derivative **F397**, SR = 2.1 for **F400**, and SR = 2 for **F421**).

Since the doubling time of A549 cells is 22 h and the doubling time of A549/DX cells is 61 h (data not shown), in this first experimental set we used a 24 h time, i.e., the most suitable for A549 cells’ cytotoxicity as performed in the “pure” MRP1 model. Indeed, the accurate detection of cell viability in A549 cells was critical in calculating the SR in a reliable manner. The following experiments, all performed in A549/DX cells, were performed after 72 h, i.e., the best timing to detect a cytotoxic effect on the slowly growing resistant population.

**Figure 3:**
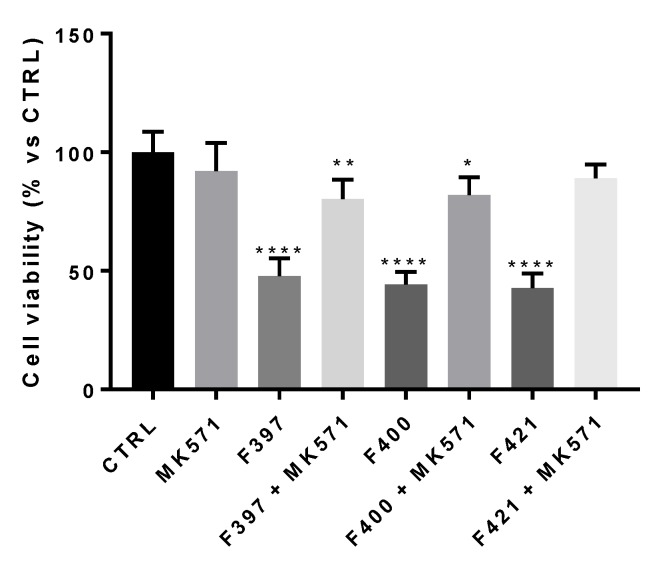
Cell viability of F397, F400, and F421 at 10 μM alone and with MK571 at 25 μM on the A549/DX cell line. Each bar represents the mean ± SEM of three experiments performed in triplicate. One-way analysis of variance (ANOVA) analysis: * *p* < 0.05; ** *p* < 0.01; **** *p* < 0.0001 vs. control.

First, we wondered if there was a correlation between the inhibition of the MRP1 protein and the CS properties of the compounds. To this aim, we adopted a concentration similar to their EC50, i.e., 10 µM. In this experimental condition, the three compounds resulted as cytotoxic, confirming their behavior as CS-promoting agents in A549/DX cells (Figure 3). 

In order to confirm that the observed effect is due to the interaction of the ligands with MRP1, the cellular viability at 72 h has been measured, testing the three ligands in co-administration with the MRP1 inhibitor, MK571, at 25 μM, a concentration that fully inhibited the MRP1 ATPase activity on A549/DX cells (ATPase activity in untreated A549/DX cells: 4.32 ± 0.58 nmoles Pi/min/mg prot; ATPase activity in MK571-treated A549/DX cells: 0.40 ± 0.12 nmoles Pi/min/mg prot). As depicted in Figure 3, the cytotoxicity of the three compounds was reverted by the MRP1 inhibitor MK571, confirming that MRP1 is the target of the **F397**, **F400,** and **F421** compounds in A549/DX.

While **F400** is devoid of sigma-2 receptor affinity [42], **F421** and **F397** respectively display a moderate (*K*_i_ = 169 nM) [41] to a high affinity (*K*_i_ = 5.34 nM) [28] towards sigma-2 receptors. Nevertheless, the involvement of a sigma-2 receptor-mediated effect was ruled out by the detection of a low density of sigma-2 receptors in A549 cells (data not shown). Additionally, besides the reversal of the activity of these ligands by the MRP1 inhibitor MK571, worthy of note is the result which was obtained with the three sensitizers in the MCF7 cell line, having a high level of sigma-2 and low level of MRP1 (as depicted in Figure 1). While **F400** and **F421** were not cytotoxic in these cells (EC_50_ > 100 μM, data not shown), **F397** showed an EC_50_ = 17.8 μM [28]. Importantly, cytotoxic activity of **F397**, which was shown to be a potent P-gp inhibitor in the MCF7 cell line (EC_50_ = 0.21 μM), was lower in the corresponding resistant MCF7/DX overexpressing P-gp (EC_50_ = 21.8 μM) [28]. These data strongly support the MRP1-mediated CS action of **F397** (results from A549 and MDCK cells and their resistant counterparts that overexpress MRP1 but not P-gp), that by contrast, is devoid of P-gp-mediated CS properties (results from MCF7 cells).

We next observed the antiproliferative effect of these three compounds co-administrated with the elective drug usually chosen for the treatment of the non-small cell lung cancer, cis-platinum (cis-Pt). Cis-Pt is effective against cancer cells because it creates cross-links between purine bases, alters the double helix conformation, and induces DNA strand breaks, impairing the DNA repairing machinery as well. These DNA damages trigger apoptosis in sensitive cells [50]. For this broad spectrum of activities, cis-Pt is the first line of treatment in several cancer types, including lung cancer [50]. Unfortunately, since cis-Pt is a substrate of MRP1 [1], which effluxes cis-Pt, limiting its intracellular accumulation, MRP1-expressing cancer cells are usually resistant to the cytotoxic effect of the drug [51,52]. This is the case of A459/DX cells, rich with MRP1, a prototype of lung cancer cells resistant to cis-Pt. 

**Figure 4:**
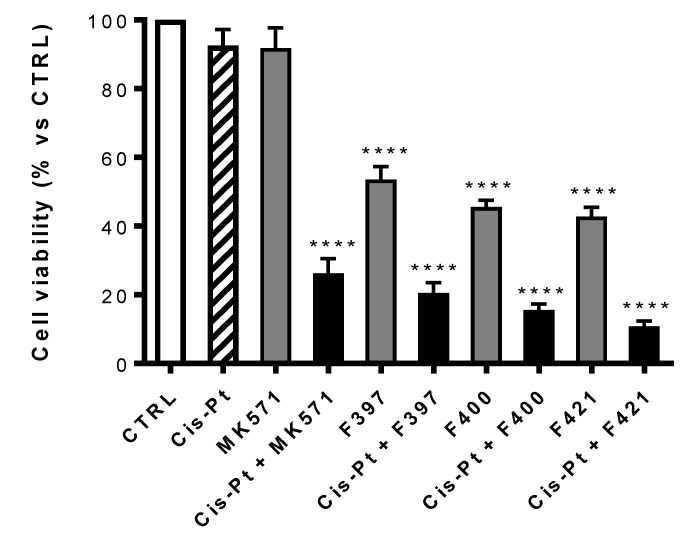
Antiproliferative activity on A549/DX of MK571 and cis-Pt at 25 μM alone and in co-administration; cis-Pt at 25 μM and respectively F397, F400, and F421 at 10 μM alone and in co-administration. Each bar represents the mean ± SEM of three experiments performed in triplicate. One-way analysis of variance (ANOVA) analysis: **** *p* < 0.0001 vs. control.

At first, the viability of each compound on A549/DX after 72 h treatment, alone or in combination with the MRP1 inhibitor MK571 or the antineoplastic drug cis-Pt both at 25 μM has been evaluated. The concentration of cis-Pt at 25 μM was chosen as A549/DX cells are platinum-resistant [53], but cells are re-sensitized when the anticancer drug is co-administered with MK571 at the same concentration. As illustrated in Figure 4, MK571 alone was not cytotoxic to A549/DX cells. The sensitivity to cis-Pt was restored when co-administrated with MK571, suggesting that MRP1 plays a key role in inducing the resistance to cis-Pt in our model. 

Using MK571 as a reference, a comparison was made between the cytotoxic effect of the compounds **F397**, **F400,** and **F421** alone at 10 μM and in association with cis-Pt at 25 μM (Figure 4). All the three compounds have a cytotoxic effect on their own, that drastically increases upon co-administration with cis-Pt. 

**Figure 5:**
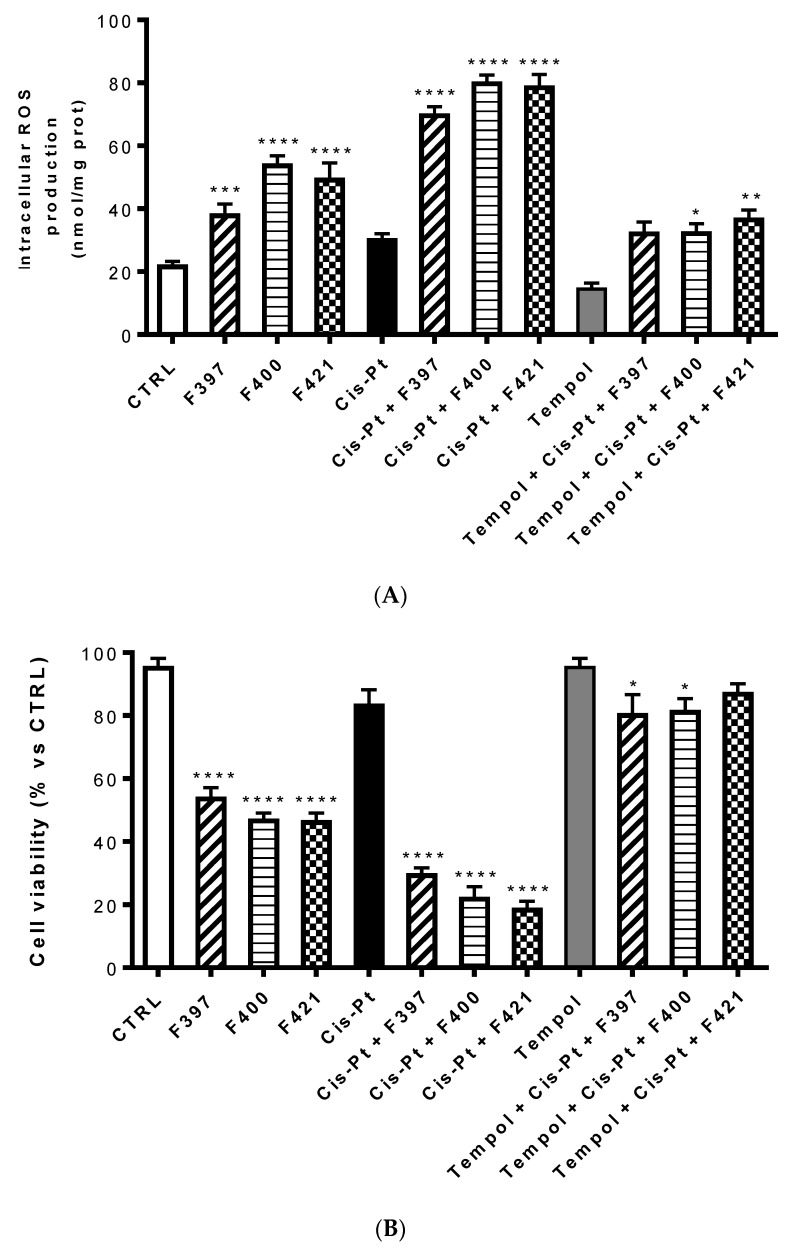
Intracellular ROS production at 24 h (**A**) and cell viability at 72 h (**B**) of compounds **F397**, **F400,** and **F421** alone at 10 μM and with 25 μM cis-Pt and/or 10 mM Tempol. Each bar represents the mean ± SEM of three experiments performed in triplicate. One-way analysis of variance (ANOVA) analysis: * *p* < 0.05; ** *p* < 0.01; **** *p* < 0.0001 vs. control.

One of the possible reasons of this sensitization is the ability of these three compounds to inhibit the efflux of cis-Pt, which is MRP1-mediated. Interestingly, the cytotoxic effect of the three compounds combined with cis-Pt was slightly more marked than the effect of MK571 in terms of cell viability. This could be associated with a second reason, considering one of the putative mechanisms of CS consisting of an increased production of ROS, capable of triggering the apoptosis of MDR cells. To investigate whether this mechanism is involved in the sensitizing effects elicited by **F397**, **F400,** and **F421**, we measured the intracellular ROS production in A549/DX after 24 h treatment with these three molecules at 10 μM, with and without 25 μM cis-Pt (Figure 5A). We noticed that these three compounds alone increased the amount of intracellular ROS. This was further enhanced when they were co-administrated with the antineoplastic drug, thus allowing its antitumor activity. As reported in Figure 5B, this effect was reverted by the ROS scavenger Tempol, tested at 10 mM. Moreover, to verify whether the increase in ROS induced the reduction in cell viability elicited by the compounds, we evaluated the antiproliferative effect at 72 h of these three sensitizers alone and with 25 μM cis-Pt, adding 10 mM Tempol at time zero and after 48 h from the incubation. As shown in Figure 5B, the data obtained are in line with the ROS production hypothesis: higher were the ROS levels, lower was cell viability, and vice-versa. 

**Figure 6:**
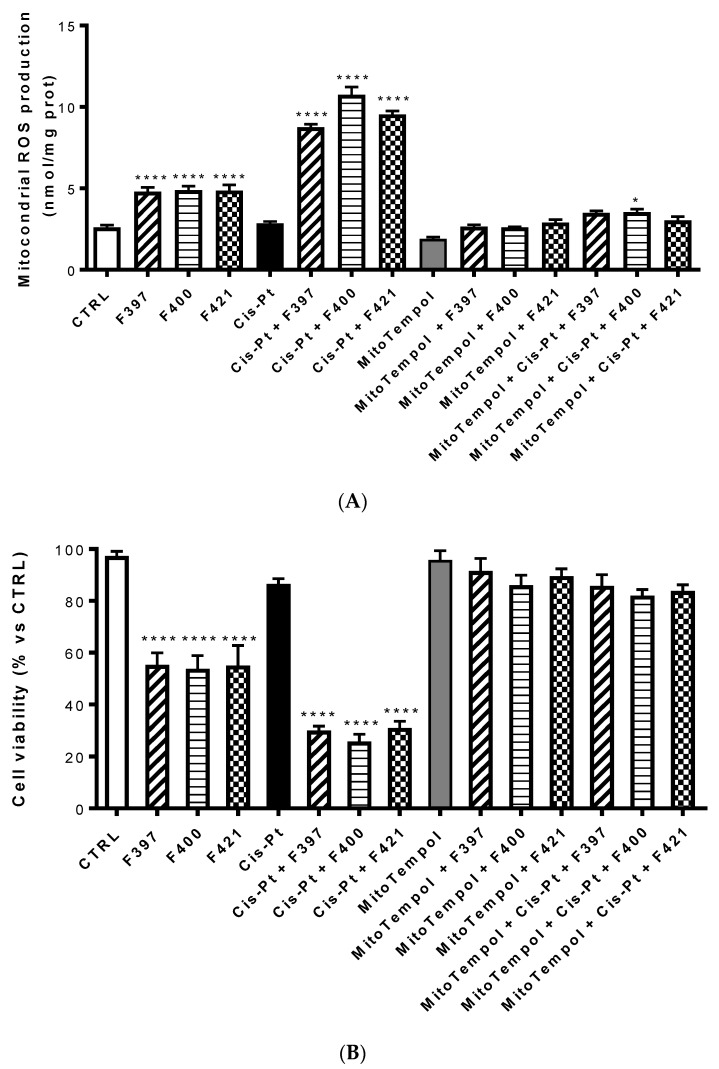
Mitochondrial ROS production (**A**) and the relative cellular vitality (**B**) on A549/DX at 24 h using Mitotempol as the scavenger of the mithocondrial ROS in the presence of **F397**, **F400**, and **F421**, alone at 10 μM, and in the presence of Mitotempol (10 mM) and cis-platinum (25 μM). Each bar represents the mean ± SEM of three experiments performed in triplicate. One-way analysis of variance (ANOVA) analysis: **** *p* < 0.0001, * *p* < 0.05 vs. control.

An increase in ROS of mitochondrial origin is particularly effective in killing multidrug-resistant cells [53]. To investigate whether the ROS were of mithocondrial origin, the same experiment was set up evaluating ROS production at 24 h with Mitotempol, the scavenger of mithocondrial ROS. As illustrated in Figure 6A, compounds **F327**, **F400,** and **F421** increased the intramithocondrial ROS. This effect was enforced by the concurrent administration of cis-Pt, but reversed by Mitotempol. Evaluating the relative cellular vitality on A549/DX at 72 h, the antiproliferative effect of cis-Pt, negligible when the drug was used alone, was enhanced by the CS-promoting agents and reverted by Mitotempol (Figure 6B). 

### 2.3. In Vivo Tumor Growth 

**Figure 7:**
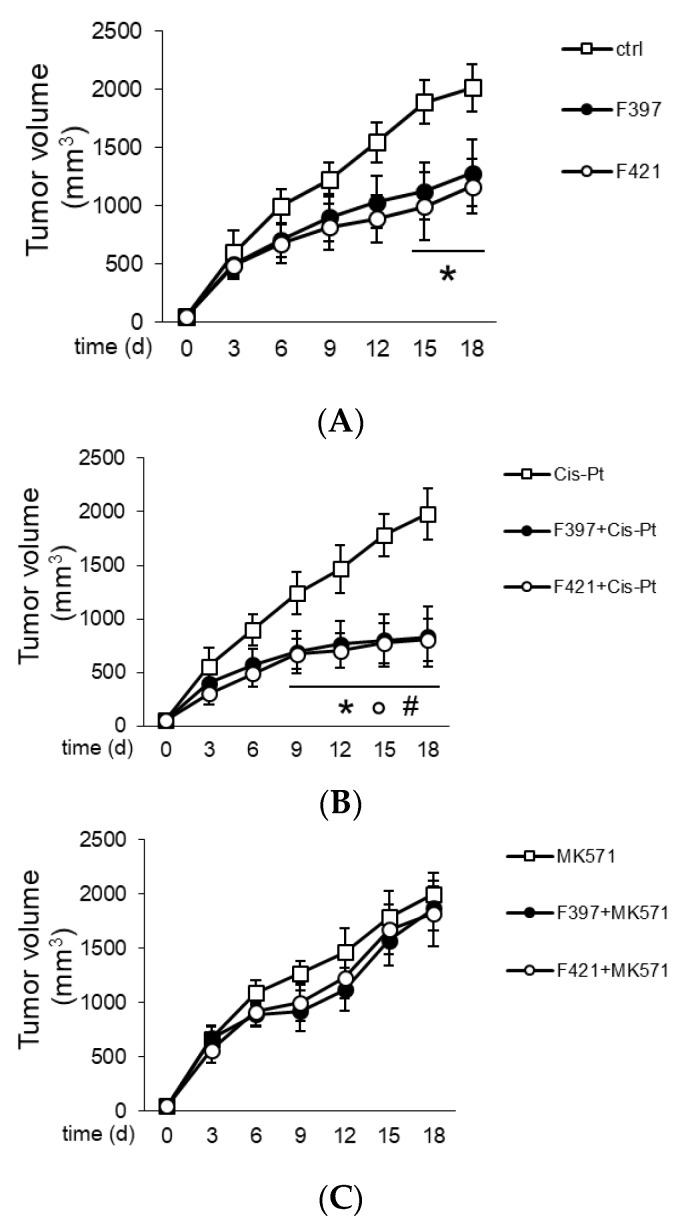
Tumor growth curves: (**A**) Treatment with saline buffer (ctrl), **F397** and **F421** at 7.5 nM; (**B**) administration of 50 mg/Kg cis-Pt alone and with two target compounds **F397** or **F421**; (**C**) administration of 3 mg/Kg MK571, the MRP1 inhibitor, alone and with **F397** and **F421**.

In order to translate in vivo the capability of **F397**, **F400**, and **F421** to act as “sensitizers”, A549/DX cells were implanted in immune-deficient BALB/C mice, and once the tumor reached the volume of 50 mm^3^, the mice were randomized and treated three times weekly with the vehicle (saline solution) and/or cis-Pt. As depicted in Figure 7, the tumor volume was monitored daily, confirming the resistance of A549/DX against the standard chemotherapy regimen. 

The animals were treated for three times weekly with a single dose of the indole-based ligands **F397** or **F421**, while **F400** could not be dissolved in solvents suitable for in vivo studies and could not be tested in vivo. The administration of **F397** or **F421** as single agents determined a low growth reduction (Figure 7A), but their co-administration with cis-Pt greatly reduced tumor growth (Figure 7B). As for the in vitro experiments, co-administration of the MRP1 inhibitor MK571 with these sensitizers abated the cytotoxic effect, supporting that the effect of **F397** and **F421** is MRP1-mediated (Figure 7C). Of note, the dose of **F397** and **F421** effective as an anti-tumor agent in vivo was lower than the dosage effective as an anti-proliferative agent in vitro. One possible reason could be that part of the anti-tumor effect of the compounds was mediated by their activity on the tumor microenvironment, not necessarily only on tumor cells. This aspect cannot be evaluated in the viability assays performed in vitro and could lead to an underestimation of the compounds’ potency. Further, the repeated administration of **F397** and **F421** followed in vivo instead of the single dosage used in vitro can be an additional explanation. Indeed, repeated and lower doses of anti-tumor agents have been reported to be more effective than one single higher dosage against chemoresistant tumors [54]. This could also be the case of **F397** and **F421** against A549/DX tumors. Notably, the reduction of cell proliferation in vitro (i.e., 50%) is similar to the extent of tumor decreases observed in vivo, indicating a good correlation between the two experimental settings. It is also worth noting that the treatment was not toxic for liver, heart, and kidney compared with the control, as shown from the hemato-chemical parameters of the animals (Table 2).

## 3. Experimental Section

### 3.1. Materials

Compounds **F397**, **F421**, and **F400** were obtained according to the previously reported procedures [27,42,43]. Cell culture reagents were purchased from Celbio s.r.l. (Milano, Italy). CulturePlate 96/well plates were purchased from PerkinElmer Life Science; Calcein-AM were obtained from Sigma-Aldrich (Milan, Italy).

### 3.2. Cell Cultures

MDCK and MDCK-MRP1 cells were a gift of Prof. P. Borst, NKI-AVL Institute, Amsterdam, The Netherlands. The MDCK cells were grown in DMEM high glucose supplemented with a 10% fetal bovine serum, 2 mM glutamine, 100 U/mL penicillin, and 100 μg/mL streptomycin in a humidified incubator at 37 °C with a 5% CO_2_ atmosphere. 

### 3.3. Cancer Cell Lines

Human colon cancer HT29 cells and their doxorubicin-resistant counterpart HT29/DX, human non-small cell lung cancer A549 cells and their doxorubicin-resistant counterpart A549/DX, and human breast cancer MCF7, SKBR3, T74D and MDA-MB-231 cells were purchased from American Type Culture Collection (ATCC; Manassas, VA, USA). The resistant sublines HT29/DX and A549/DX were obtained by culturing cells in a medium containing increasing concentrations of doxorubicin. Every 5 passages, the concentration of doxorubicin was increased according to this protocol: 100 pM (passages 1–5), 250 pM (passages 6–10), 500 pM (passages 11–15), 1 nM (passages 16–20), 25 nM (passages 21–25), 50 nM (passages 26–30), and 100 nM (passages 31–35). HT29/DX cells were used between passages 32 and 35, and were maintained in a medium with 100 nM doxorubicin, added three times/week, 6 h after cell seeding. A549/DX cells were used between passages 26 and 30, and were maintained in a medium with 50 nM doxorubicin, added three times/week, 6 h after cell seeding. In these experimental conditions, both cell lines displayed cross-resistance to different drugs, including platinum salts [44,45]. HT29, HT29/DX, MCF7, SKBR3, T74D, and MDA-MB-231 cells were cultured in an RPMI-1640 medium, and A549 and A549/DX cells were cultured in a HAM-F12 medium, containing a 10% fetal bovine serum and 1% penicillin-streptomycin, in a humidified atmosphere at 37°C. All cells were used before passage 10.

### 3.4. Calcein-AM Experiments

These experiments were carried out as described by Riganti et al., with minor modifications [55]. Each cell line (30,000 cells per well) was seeded into a black CulturePlate 96/well plate with a 100 μL medium and allowed to become confluent overnight. The 100 μL of test compounds was solubilized in a culture medium and added to monolayers, with final concentrations ranging from 0.1 to 100 μM. The 96/well plates were incubated at 37 °C for 30 min. Calcein-AM was added in 100 μL of phosphate buffered saline (PBS) to yield a final concentration of 2.5 μM, and then the plates were incubated for 30 min. Each well was washed 3 times with ice-cold PBS. Saline buffer was added to each well and the plates were read with the Victor3 plate reader (PerkinElmer) at excitation and emission wavelengths of 485 and 535 nm, respectively. In these experimental conditions, the Calcein cell accumulation in the absence and in the presence of the tested compounds was evaluated and the fluorescence basal level was estimated with the untreated cells. In treated wells, the increase in fluorescence with respect to the basal level was measured. EC_50_ values were determined by fitting the fluorescence increase percentage vs. log(dose).

### 3.5. Antiproliferative Assay

The determination of cell growth was performed using an MTT assay at 24 or 72 h [55]. On day 1, 10,000 cells/well were seeded into 96-well plates in a volume of 100 μL. On day 2, the drugs concentrations (0.1, 1, 10, 25 μM) were added. In all the experiments, the various drug solvents (ethanol, DMSO) were added in each control to evaluate a possible solvent cytotoxicity. After the established incubation time with drugs, 10 μL MTT (0.5 mg/mL) was added to each well, and after 3 h incubation at 37°C, the supernatant was removed. The formazan crystals were solubilized using 100 μL of DMSO and the absorbance values at 570 and 630 nm were determined on the microplate reader Victor 3 from PerkinElmer Life Sciences. The absorbance of the untreated cells was considered equal to 100% cell viability; the viability of cells measured in each experimental condition was expressed as a percentage of viable cells in the considered condition vs. the viability of the untreated cells.

### 3.6. Immunoblotting

Cells were rinsed with a boiling 0.5 mL lysis buffer (10 mM Tris, 100 mM NaCl, 20 mM KH_2_PO_4_, 30 mM EDTA, 1 mM EGTA, 250 mM sucrose; pH 7.5). After sonication, 1 mM Na_3_VO_4_, 1 mM NaF, 10 mM dithiothreitol, and the inhibitor cocktail set III (100 mM 4-(2-aminoethyl)benzenesulfonyl fluoride, 80 mM aprotinin, 5 mM bestatin, 1.5 mM E-64, 2 mM leupeptin, 1 mM pepstatin; Calbiochem, San Diego, CA, USA) were added and cell lysates were centrifuged at 13,000× *g* for 15 min. Then, 50 μg cell proteins were separated by SDS-PAGE and probed with an anti-MRP1/ABCC1 (IU2H10, Abcam, Cambridge, UK) and anti-actin (A2066, Sigma Chemical Co.) antibody, used as the control of equal loading. Immunoblot quantitation (band density ratio MRP1/actin) was performed using the ImageJ software (https://imagej.nih.gov/ij/). 

### 3.7. Glutathione Measurement

Cells were washed with PBS and 600 µL 0.01 N HCl was added. After gentle scraping, cells were frozen/thawed twice and proteins were precipitated by adding 120 µL of 6.5% w/v 5-sulfosalicylic acid to 480 µL of lysate. Each sample was placed in ice for 1 h and centrifuged for 15 min at 13,000× *g* (4 °C). The protein content was measured with a BAC Kit (Sigma Chemicals. Co), as per the manufacturer’s instruction. Total glutathione was measured in 20 µL of the cell lysate or supernatant with the following reaction mix: 20 µL of stock buffer (143 mM NaH_2_PO_4_ and 63 mM EDTA, pH 7.4), 200 µL of daily reagent (10 mM 5,5’dithiobis-2-nitrobenzoic acid and 2 mM NADPH in stock buffer), and 40 µL of glutathione reductase (8.5 U/mL). The content of the oxidized glutathione (GSSG) was obtained after the derivatization of GSH with 2-vinylpyridine (2VP): 10 µL of 2VP was added to 200 µL of cell lysate or culture supernatant and the mixture was shaken at room temperature for 1 h. Glutathione was then measured in 40 µL of the sample as described. The reaction was followed kinetically for 5 min using a Synergy HT Multi-Mode Microplate Reader (Bio-Tek Instruments, Winooski, VT, USA), measuring the absorbance at 415 nm. Each measurement was made in triplicate and results were expressed as nmol of glutathione/min/mg cellular protein, according to a titration curve set up with serial dilutions (10 µM–0.1 nM) of 1:1 GSH/GSSG mix. For each sample, GSH was obtained by subtracting the GSSG from the total glutathione.

### 3.8. Total Reactive Oxygen Species (ROS) Measurement

Here, 1 × 10^6^ whole cells were re-suspended in a final volume of 0.5 mL PBS and incubated for 30 min at 37 °C with 5 μM of the fluorescent probe 5-(and-6)-chloromethyl-2’,7’-dichlorodihydro-fluorescein diacetate-acetoxymethyl ester (DCFDA-AM, Sigma Chemicals Co.) in the dark. Cells were then centrifuged at 13,000× *g* at 37 °C and re-suspended in 0.5 mL PBS. The fluorescence of each sample, considered as the index of ROS levels, was read at 492 (λ excitation) and 517 nm (λ emission) using a Synergy HT Multi-Mode Microplate Reader (Bio-Tek Instruments). The results were expressed as nmol total ROS/mg cell proteins. A preliminary titration curve was set up by incubating the cells for 1 h with the cells treated with serial dilutions (10–0.01 nM) of the pro-oxidant agent menadione.

### 3.9. Mitochondrial ROS Measurement

Here, 1 × 10^6^ cells were re-suspended in a final volume of 0.5 mL PBS and incubated for 30 min at 37 °C with 5 μM of the fluorescent probe MitoSOX Red (Invitrogen Life Technology, Milano, Italy) in the dark. Cells were then centrifuged at 13,000× *g* at 37 °C and re-suspended in 0.5 mL PBS. The fluorescence of each sample, considered as the index of ROS levels, was read at 510 (λ excitation) and 580 nm (λ emission) using a Synergy HT Multi-Mode Microplate Reader (Bio-Tek Instruments). The results were expressed as nmol mitochondrial ROS/mg cell proteins. A preliminary titration curve was set up by incubating the cells for 1 h with the cells treated with serial dilutions (10–0.01 nM) of the pro-oxidant agent menadione.

### 3.10. MRP1 Activity

Cells were washed with Ringer’s solution (148.7 mM NaCl, 2.55 mM K_2_HPO_4_, 0.45 mM KH_2_PO_4_, 1.2 mM MgSO_4_; pH 7.4), lysed on crushed ice with lysis buffer (10 mM Hepes/Tris, 5 mM EDTA, 5 mM EGTA, 2 mM dithiothreitol; pH 7.4) supplemented with 2 mM PMSF, 1 mmol/L aprotinin, 10 μg/mL pepstatin, and 10 μg/mL leupeptin, and subjected to nitrogen cavitation at 1200 psi for 20 min. Samples were centrifuged at 300× *g* for 10 min, overlaid on a sucrose cushion (10 mM Tris/HCl, 35% w/v sucrose, 1 mM EDTA; pH 7.5) and centrifuged at 14,000× *g* for 10 min. The interface was collected, re-suspended in the centrifugation buffer (10 mM Tris/HCl, 250 mM sucrose; pH 7.5) and subjected to a third centrifugation at 100,000× *g* for 45 min. The vesicle pellet was re-suspended in a 0.5 mL centrifugation buffer and used for the protein quantification using the BCA kit (Sigma-Merck). Then, 100 μg of the proteins were immuno-precipitated in 100 μL of the centrifugation buffer overnight at 4 °C with an anti-MRP1 antibody (diluted 1:50, Abcam, Cambridge, MA), using 25 µL pure proteome beads A/G (Merck/Millipore, Burlington, MA, USA), and then re-suspended in 50 μL of the centrifugation buffer. Samples were incubated for 30 min at 37 °C with 50 μL of the reaction mix (25 mM Tris/HCl, 3 mM ATP, 50 mM KCl, 2.5 mM MgSO_4_, 3 mM dithiothreitol, 0.5 mM EGTA, 2 mM ouabain, 3 mM NaN_3_; pH 7.0). The reaction was stopped by adding 0.2 mL ice-cold stopping buffer (0.2% w/v ammonium molybdate, 1.3% v/v H_2_SO_4_, 0.9% w/v SDS, 2.3% w/v trichloroacetic acid, 1% w/v ascorbic acid). After a 30-min incubation at room temperature, the absorbance of the phosphate hydrolyzed from ATP was measured at 620 nm, using a Synergy HT Multi-Mode Microplate Reader (Bio-Tek Instruments). The absorbance was converted into μmol hydrolyzed phosphate/min/mg proteins, according to the titration curve previously prepared with serial dilutions (100–0.1 nM) of NaHPO_4_.

### 3.11. In Vivo Tumor Growth

Here, 1 × 10^6^ A549/DX cells were mixed with 100 μl Matrigel implanted in 6-week-old female nu/nu BALB/C mice (Charles River Laboratories Italia, Calco, Italy), housed (5 per cage) under 12 h light/dark cycle, with food and drinking provided ad libitum. Tumor growth was measured daily by caliper, according to the equation (LxW2)/2, where L = tumor length and W = tumor width. When the tumor reached the volume of 50 mm^3^, mice (*n* = 8/group) were randomized and treated as reported in the following groups, treated on day 1, 7, and 14 after randomization: (1) vehicle group (ctrl), treated intraperitoneally (i.p.) with 100 µL saline solution; (2) cis-Pt group, treated i.p. with 50 mg/kg cis-Pt, dissolved in 100 µL water/10% DMSO solution, according to Kopecka et al., 2015 [53]; (3) **F397** group, treated i.p. with 750 nmoles of the compound, dissolved in 100 µL water/10% DMSO solution, corresponding to the maximum tolerated dose (not shown and Table 2); (4) **F421** group, treated i.p. with 750 nmoles of the compound, dissolved in 100 µL water/10% DMSO solution i.p, corresponding to the maximum tolerated dose (not shown and Table 2); (5) cis-Pt/**F397** group, treated i.p. with 50 mg/kg Cis-Pt and 750 nmoles of F397; (6) cis-Pt/**F421** group, treated i.p. with 50 mg/kg cis-Pt and 750 nmoles of F421; (7) MK571 group, treated i.p. with 3 mg/kg MK571 dissolved in 100 µL water/10% DMSO solution, according to Jones et al., 1989 [56]; (8) MK571/**F397** group, treated i.p. with 3 mg/kg MK571 and and 750 nmoles of **F397**; (9) MK571/**F421** group, treated i.p. with 3 mg/kg MK571 and 750 nmoles of **F421**. Tumor volumes were monitored daily. Animals were euthanized at day 21 after randomization with zolazepam (0.2 mL/kg) and xylazine (16 mg/kg). Lactate dehydrogenase (LDH), aspartate aminotransferase (AST), alanine aminotransferase (ALT), alkaline phosphatase (AP), creatinine, and creatine phosphokinase (CPK) were measured on blood samples collected immediately after euthanasia, using commercially available kits from Beckman Coulter Inc. (Beckman Coulter, Miami, FL, USA). 

The animal care and experimental procedures were approved by the Bio-Ethical Committee of the Italian Ministry of Health (#122/2015-PR).

### 3.12. Statistical Analysis

All data in the text and figures are provided as means ± SD. The results were analyzed by a Student’s t-test and ANOVA test, using Graph-Pad Prism (Graph-Pad software, San Diego, CA, USA). *p* < 0.05 was considered significant. The investigators responsible for the data analysis were unaware of the experimental conditions analyzed. 

## 4. Conclusions

CS, that is, a sort of synthetic lethality according to which resistant tumor cells are selectively killed (rather than their wild type counterparts), appears as a promising therapeutic approach for the treatment of resistant tumors. In this context, MDR pump modulators may be endowed with CS properties. Besides the deeply explored P-gp-mediated CS, the less explored MRP1-mediated CS has an important role. With the aim of identifying promising MRP1 modulators as CS inducers, we screened a library of sigma-2 receptor ligands which were previously identified as P-gp-mediated CS inducers. Three compounds, **F397**, **F400** and **F421**, that modulated MRP1 and displayed cytotoxicity in a number of cell lines, exerted a more potent cytotoxicity in the MRP1 overexpressing cells (MDCK/MRP1 and A549/DX) compared with the wild type counterparts, thus showing important CS properties. All the three compounds were found to alter the GSH/GSSG ratio in the cell lines studied, and to increase the mitochondrial ROS. The three compounds, that were able to exert cytotoxicity by themselves, potently synergized with cis-Pt, re-activating this “traditional” antitumor drug in cells refractory to the drug because of the MRP1 expression. These in vitro results were translated in the corresponding in vivo model of cis-Pt-resistant A549/DX xenografts, where the co-administration of the MRP1-mediated sensitizers **F397** and **F421** with cis-Pt greatly reduced tumor growth, with no signs of toxicity. Importantly, both in vitro and in vivo, the activity of these sensitizers was demonstrated to be mediated by the interaction with MRP1. Overall, we demonstrated that MRP1-mediated CS is a promising approach for the treatment of resistant tumors such as the non-small cell lung tumor, whose bad prognosis urgently requires novel therapeutic approaches. The results herein obtained make these classes of compounds worthy to be further investigated for other MRP1 overexpressing resistant tumors, while **F397**, **F400,** and **F421** may be considered as first in class for the development of novel MRP1-mediated collateral sensitizers.

## Figures and Tables

**Table 1:** MRP1 activity and collateral sensitivity activity (selectivity ratio, SR) of the three compounds **F397**, **F400**, and **F421**, and the two reference compounds verapamil and siramesine.

Compound	MRP1, EC_50_ ± SEM, μM	SR
**F397**	16.7 ± 3.2	3.41
**F400**	17.6 ± 3.8	5.91
**F421**	28 ± 4.8	2.47
Verapamil	7.9 ± 1.7	4.7
Siramesine	23.2 ± 4.5	0.56

**Table 2:** Hemato-chemical parameters of animals.

	CTRL	F397	F421	Cis-Pt	F397 + Cis-Pt	F421 + Cis-Pt	MK571	F397 + MK571	F421 + MK571
LDH (U/L)	6782 ± 567	6729 ± 492	7182 ± 892	7092 ± 542	7189 ± 823	6652 ± 629	7141 ± 927	6981 ± 554	7089 ± 504
AST (U/L)	76 ± 32	81 ± 24	82 ± 37	91 ± 34	74 ± 34	98 ± 82	101 ± 33	72 ± 32	78 ± 38
ALT (U/L)	32 ± 10	37 ± 108	33 ± 13	41 ± 9	33 ± 10	32 ± 9	41 ± 7	40 ± 11	41 ± 8
AP (U/L)	145 ± 32	112 ± 32	143 ± 34	129 ± 14	142 ± 33	132 ± 34	117 ± 33	103 ± 42	113 ± 31
Creatinine (mg/L)	0.034 ± 0.007	0.043 ± 0.009	0.045 ± 0.011	0.038 ± 0.011	0.041 ± 0.007	0.044 ± 0.012	0.044 ± 0.007	0.037 ± 0.009	0.034 ± 0.008
CPK (U/L)	298 ± 73	324 ± 72	321 ± 72	259 ± 98	301 ± 75	278 ± 65	334 ± 81	298 ± 63	292 ± 77

Balb/C mice (*n* = 8 animals/group) were treated as described. Blood was collected immediately after euthanasia and analyzed for lactate dehydrogenase (LDH), aspartate aminotransferase (AST), alanine aminotransferase (ALT), alkaline phosphatase (AP), creatinine, and creatine phosphokinase (CPK). Data are presented as means ± SD. * *p* < 0.05: vs. ctrl group.

## Abbreviations

ABC	ATP Binding Cassette
ALT	alanine aminotransferase
AP	alkaline phosphatase
AST	aspartate aminotransferase
BCRP	Breast Cancer Resistance Protein
BHK-21	Baby Hamster Kidney
Cis-Pt	Cis-platinum
CS	collateral sensitivity
CPK	creatine phosphokinase
GSH	glutathione
GSSG	oxidized glutathione
LDH	Lactate dehydrogenase
MDR	Multidrug Resistance
MRP1	Multidrug resistance-associated Protein 1
PBS	Phosphate Buffered Saline
P-gp	P glycoprotein
ROS	reactive oxygen species
SR	selectivity ratio

## References

[1] Gottesman M.M., Fojo T., Bates S.E. (2002). Multidrug resistance in cancer: Role of ATP-dependent transporters. Nat. Rev. Cancer.

[2] Colabufo N.A., Berardi F., Cantore M., Contino M., Inglese C., Niso M., Perrone R. (2010). Perspectives of P-glycoprotein modulating agents in oncology and neurodegenerative diseases: Pharmaceutical, biological and diagnostic potentials. J. Med. Chem..

[3] Riganti C., Mini E., Nobili S. (2015). Editorial: Multidrug resistance in cancer: Pharmacological strategies from basic research to clinical issues. Front. Oncol..

[4] Deeley R.G., Westlake C., Cole S.P. (2006). Transmembrane transport of endo- and xenobiotics by mammalian ATP-binding cassette multidrug resistance proteins. Physiol. Rev..

[5] Linton K.J. (2007). Structure and function of ABC transporters. Physiology (Bethesda).

[6] Gottesman M.M., Ambudkar S.V. (2001). Overview: ABC transporters and human disease. J. Bioenerg Biomembr..

[7] Colabufo N.A., Berardi F., Contino M., Niso M., Perrone R. (2009). ABC pumps and their role in active drug transport. Curr. Top. Med. Chem..

[8] Eckford P.D.W., Sharom F.J. (2009). ABC efflux pump-based resistance to chemotherapy drugs. Chem. Rev..

[9] Krishna R., Mayer L.D. (2000). Multidrug resistance (MDR) in cancer. Mechanisms, reversal using modulators of MDR and the role of MDR modulators in influencing the pharmacokinetics of anticancer drugs. Eur. J. Pharm. Sci..

[10] Salaroglio I.C., Abate C., Rolando B., Battaglia L., Gazzano E., Colombino E., Costamagna C., Annovazzi L., Mellai M., Berardi F. (2019). Validation of thiosemicarbazone compounds as P-glycoprotein inhibitors in human primary brain-blood barrier and glioblastoma stem cells. Mol. Pharm..

[11] Amiri-Kordestani L., Basseville A., Kurdziel K., Fojo A.T., Bates S.E. (2012). Targeting MDR in breast and lung cancer: Discriminating its potential importance from the failure of drug resistance reversal studies. Drug Resist. Updates.

[12] Hall M.D., Handley M.D., Gottesman M.M. (2009). Is resistance useless? Multidrug resistance and collateral sensitivity. Trends Pharmacol. Sci..

[13] Szybalski W., Bryson V. (1952). Genetic studies on microbial cross resistance to toxic agents: I. Cross resistance of Escherichia coli to fifteen antibiotics. J. Bacteriol..

[14] Pluchino K.M., Hall M.D., Goldsborough A.S., Callaghan R., Gottesman M.M. (2012). Collateral sensitivity as a strategy against cancer multidrug resistance. Drug Resist. Updates.

[15] Szakàcs G., Paterson J.K., Ludwig J.A., Booth-Genthe C., Gottesman M.M. (2006). Targeting multidrug resistance in cancer. Nat. Rev. Cancer.

[16] Szakàcs G., Hall M.D., Gottesman M.M., Boumendjel A., Kachadourian R., Day B.J., Baubichon-Cortay H., Di Pietro A. (2014). Targeting the Achilles Heel of Multidrug-Resistant Cancer by Exploiting the Fitness Cost of Resistance. Chem. Rev..

[17] Laberge R.M., Ambadipudi R., Georges E. (2009). P-glycoprotein (ABCB1) modulates collateral sensitivity of a multidrug resistant cell line to verapamil. Arch Biochem. Biophys.

[18] Cole S.P.C. (2014). Multidrug Resistance protein 1 (MRP1, ABCC1), a “Multitasking” ATP-binidng cassette (ABC) transporter. J. Boil. Chem..

[19] Lorendeau D., Dury L., Nasr R., Boumendjel A., Teodori E., Gutschow M., Falson P., Di Pietro A., Baubichon-Cortay H. (2017). MRP1-dependent collateral sensitivity of multidrug-resistant cancer cells: Identifying selective modulators inducing cellular glutathione depletion. Curr. Med. Chem..

[20] Gould N.S., Day B.J. (2011). Targeting maladaptive glutathione responses in lung disease. Biochem. Pharmacol..

[21] Perrotton T., Trompier D., Chang X.B., Di Pietro A., Baubichon-Cortay H. (2007). (R)- and (S)-verapamil differentially modulate the multidrug-resistant protein MRP1. J. Biol. Chem..

[22] Lu J.F., Pokharel D., Bebawy M. (2015). MRP1 and its role in anticancer drug resistance. Drug Metab. Rev..

[23] Qian Y.M., Song W.C., Cui H., Cole S.P., Deeley R.G. (2001). Glutathione stimulates sulfated estrogen transport by multidrug resistance protein 1. J. Biol. Chem..

[24] Leier I., Jedlitschky G., Buchholz U., Center M., Cole S.P., Deeley R.G., Keppler D. (1996). ATP-dependent glutathione disulphide transport by the MRP gene-encoded conjugate export pump. Biochem. J..

[25] Trompier D., Chang X.B., Barattin R., du Moulinet D’Hardemare A., Di Pietro A., Baubichon-Cortay H. (2004). Verapamil and its derivative trigger apoptosis through glutathione extrusion by multidrug resistance protein MRP1. Cancer Res..

[26] Laberge R.M., Karwatsky J., Lincoln M.C., Leimanis M.L., Georges E. (2007). Modulation of GSH levels in ABCC1 expressing tumor cells triggers apoptosis through oxidative stress. Biochem. Pharmacol..

[27] Barattin R., Perrotton T., Trompier D., Lorendeau D., Pietro A.D., d’Hardemare Adu M., Baubichon-Cortay H. (2010). Iodination of verapamil for a stronger induction of death, through GSH efflux, of cancer cells overexpressing MRP1. Bioorg. Med. Chem..

[28] Niso M., Abate C., Contino M., Ferorelli S., Azzariti A., Perrone R., Colabufo N.A., Berardi F. (2013). Sigma-2 receptor agonists as possible antitumor agents in resistant tumors: Hints for collateral sensitivity. Chem. Med. Chem..

[29] Niso M., Riganti C., Abate C. (2014). Collateral sensitivityof 2 ligands: Potentials in the treatment of multidrug resistant tumors. Recept. Clin. Investig..

[30] Pape V.F.S., Toth S., Füredi A., Szebenyi K., Lovrics A., Szabo P., Wiese M., Szakacs G. (2016). Design, synthesis and biological evaluation of thiosemicarbazones, hydrazinobenzothiazoles and arylhydrazones as anticancer agents with a potential to overcome multidrug resistance. Eur. J. Med. Chem..

[31] Gana C.C., Hanssen K.M., Yu D.M.T., Flemming C.L., Wheatley M.S., Conseil G., Cole S.P.C., Norris M.D., Haber M., Fletcher J.I. (2019). MRP1 modulators synergize with buthionine sulfoximine to exploit collateral sensitivity and selectively kill MRP1-expressing cancer cells. Biochem. Pharmacol..

[32] Tan K.W., Sampson A., Osa-Andrews B., Iram S.H. (2018). Calcitriol and Calcipotriol Modulate Transport Activity of ABC Transporters and Exhibit Selective Cytotoxicity in MRP1-overexpressing Cells. Drug Metab. Dispos..

[33] Pati M.L., Niso M., Ferorelli S., Abate C., Berardi F. (2015). Novel metal chelators thiosemicarbazones with activity at the σ2 receptors and P-glycoprotein: An innovative strategy for resistant tumor treatment. RSC Adv..

[34] Pati M.L., Niso M., Spitzer D., Berardi F., Contino M., Riganti C., Hawkins W.G., Abate C. (2018). Multifunctional thiosemicarbazones and deconstructed analogues as a strategy to study the involvement of metal chelation, Sigma-2 (σ2) receptor and P-gp protein in the cytotoxic action: In vitro and in vivo activity in pancreatic tumors. Eur. J. Med. Chem..

[35] Alon A., Schmidt H.R., Wood M.D., Sahn J.J., Martin S.F., Kruse A.C. (2017). Identification of the gene that codes for the σ2 receptor. PNAS.

[36] Abate C., Niso M., Berardi F. (2018). Sigma-2 receptor: Past, present and perspectives on multiple therapeutic exploitations. Future Med. Chem..

[37] Schmidt H.R., Kruse A.C. (2019). The Molecular Function of σ Receptors: Past, Present, and Future. Trends Pharmacol. Sci..

[38] Cantonero C., Camello P.J., Abate C., Berardi F., Salido G.M., Rosado J.A., Redondo P.C. (2020). NO1, a New Sigma 2 Receptor/TMEM97 Fluorescent Ligand, Downregulates SOCE and Promotes Apoptosis in the Triple Negative Breast Cancer Cell Lines. Cancers.

[39] Liu C.C., Yu C.F., Wang S.C., Li H.Y., Lin C.M., Wang H.H., Abate C., Chiang C.S. (2019). Sigma-2 receptor/TMEM97 agonist PB221 as an alternative drug for brain tumor. BMC Cancer.

[40] Pati M.L., Hornick J.R., Niso M., Berardi F., Spitzer D., Abate C., Hawkins W. (2017). Sigma-2 receptor agonist derivatives of 1-Cyclohexyl-4-[3-(5-methoxy-1,2,3,4-tetrahydronaphthalen-1-yl)propyl]piperazine (PB28) induce cell death via mitochondrial superoxide production and caspase activation in pancreatic cancer. BMC Cancer.

[41] Pati M.L., Abate C., Contino M., Ferorelli S., Luisi R., Carroccia L., Niso M., Berardi F. (2015). Deconstruction of 6,7-dimethoxy-1,2,3,4-tetrahydroisoquinoline moiety to separate P-glycoprotein (P-gp) activity from σ2 receptor affinity in mixed P-gp/σ2 receptor agents. Eur. J. Med. Chem..

[42] Abate C., Pati M.L., Contino M., Colabufo N.A., Perrone R., Niso M., Berardi F. (2015). From mixed sigma-2 receptor/P-glycoprotein targeting agents to selective P-glycoprotein modulators: Small structural changes address the mechanism of interaction at the efflux pump. Eur. J. Med. Chem..

[43] Perregaard J., Moltzen E.K., Meier E., Sánchez C. (1995). Sigma ligands with subnanomolar affinity and preference for the sigma 2 binding site. 1. 3-(omega-aminoalkyl)-1*H*-indoles. J. Med. Chem..

[44] Ostenfeld M.S., Fehrenbacher N., Høyer-Hansen M., Thomsen C., Farkas T., Jäättelä M. (2005). Effective tumor cell death by sigma-2 receptor ligand siramesine involves lysosomal leakage and oxidative stress. Cancer Res..

[45] Abate C., Niso M., Lacivita E., Mosier P.D., Toscano A., Perrone R. (2011). Analogues of σ receptor ligand 1-cyclohexyl-4-[3-(5-methoxy-1,2,3,4-tetrahydronaphthalen-1-yl)propyl]piperazine (PB28) with added polar functionality and reduced lipophilicity for potential use as positron emission tomography radiotracers. J. Med. Chem..

[46] Kweon M.H., Adhami V.M., Lee J.S., Mukhtar H. (2006). Constitutive overexpression of Nrf2-dependent heme oxygenase-1 in A549 cells contributes to resistance to apoptosis induced by epigallocatechin 3-gallate. J. Biol. Chem..

[47] Polimeni M., Voena C., Kopecka J., Riganti C., Pescarmona G., Bosia A., Ghigo D. (2011). Modulation of doxorubicin resistance by the glucose-6-phosphate dehydrogenase activity. Biochem. J..

[48] Giacomini I., Ragazzi E., Pasut G., Montopoli M. (2020). The Pentose Phosphate Pathway and Its Involvement in Cisplatin Resistance. Int. J. Mol. Sci..

[49] Lan D., Wang L., He R., Ma J., Bin Y., Chi X., Chen G., Cai Z. (2018). Exogenous glutathione contributes to cisplatin resistance in lung cancer A549 cells. Am. J. Transl. Res..

[50] Dasari S., Tchounwou P.B. (2014). Cisplatin in cancer therapy: Molecular mechanisms of action. Eur. J. Pharmacol..

[51] Riganti C., Kopecka J., Panada E., Barak S., Rubinstein M. (2015). The role of C/EBP-β LIPi n multidrug resistance. J. Natl. Cancer Inst..

[52] Fang Z., Chen W., Yuan Z., Liu X., Jiang H. (2018). LncRNA-MALAT1 contributes to the cisplatin-resistance of lung cancer by upregulating MRP1 and MDR1 via STAT3 activation. Biomed. Pharmacother..

[53] Kopecka J., Porto S., Lusa S., Gazzano E., Salzano G., Giordano A., Desiderio V., Ghigo D., Caraglia M., De Rosa G. (2015). Self-assembling nanoparticles encapsulating zoledronic acid revert multidrug resistance in cancer cells. Oncotarget.

[54] Riganti C., Gazzano E., Gulino G.R., Volante M., Ghigo D., Kopecka J. (2015). Two repeated low doses of doxorubicin are more effective than a single high dose against tumors overexpressing P-glycoprotein. Cancer Lett..

[55] Riganti C., Contino M., Guglielmo S., Perrone M.G., Salaroglio I.C., Milosevic V., Giampietro R., Leonetti F., Rolando B., Lazzarato L. (2019). Design, Biological Evaluation, and Molecular Modeling of Tetrahydroisoquinoline Derivatives: Discovery of A Potent P-Glycoprotein Ligand Overcoming Multidrug Resistance in Cancer Stem Cells. J. Med. Chem..

[56] Jones T.R., Zamboni R., Belley M., Champion E., Charette L., Ford-Hutchinson A.W., Frenette R., Gauthier J.Y., Leger S., Masson P. (1989). Pharmacology of L-660,711 (MK-571): A novel potent and selective leukotriene D4 receptor antagonist. Can. J. Physiol. Pharmacol..

